# Cadmium Chloride Induces DNA Damage and Apoptosis of Human Liver Carcinoma Cells via Oxidative Stress

**DOI:** 10.3390/ijerph13010088

**Published:** 2016-01-02

**Authors:** Anthony Skipper, Jennifer N. Sims, Clement G. Yedjou, Paul B. Tchounwou

**Affiliations:** 1 Molecular Toxicology Research Laboratory, NIH-Center for Environmental Health, College of Science, Engineering and Technology, Jackson State University, 1400 Lynch Street, Box 18540, Jackson, MS 39217, USA; anthony.c.skipper@gmail.com (A.S.); clement.yedjou@jsums.edu (C.G.Y.); 2 Department of Pathology, Beth Israel Deaconess Medical Center, Harvard Medical School, 330 Bookline Avenue, Boston, MA 02215, USA; jnsims@bidmc.harvard.edu

**Keywords:** cadmium chloride, HepG_2_ cells, cytotoxicity, oxidative stress, DNA damage, apoptosis

## Abstract

Cadmium is a heavy metal that has been shown to cause its toxicity in humans and animals. Many documented studies have shown that cadmium produces various genotoxic effects such as DNA damage and chromosomal aberrations. Ailments such as bone disease, renal damage, and several forms of cancer are attributed to overexposure to cadmium.  Although there have been numerous studies examining the effects of cadmium in animal models and a few case studies involving communities where cadmium contamination has occurred, its molecular mechanisms of action are not fully elucidated. In this research, we hypothesized that oxidative stress plays a key role in cadmium chloride-induced toxicity, DNA damage, and apoptosis of human liver carcinoma (HepG_2_) cells. To test our hypothesis, cell viability was determined by MTT assay. Lipid hydroperoxide content stress was estimated by lipid peroxidation assay. Genotoxic damage was tested by the means of alkaline single cell gel electrophoresis (Comet) assay. Cell apoptosis was measured by flow cytometry assessment (Annexin-V/PI assay). The result of MTT assay indicated that cadmium chloride induces toxicity to HepG_2_ cells in a concentration-dependent manner, showing a 48 hr-LD_50_ of 3.6 µg/mL. Data generated from lipid peroxidation assay resulted in a significant (*p <* 0.05) increase of hydroperoxide production, specifically at the highest concentration tested. Data obtained from the Comet assay indicated that cadmium chloride causes DNA damage in HepG_2_ cells in a concentration-dependent manner. A strong concentration-response relationship (*p* *<* 0.05) was recorded between annexin V positive cells and cadmium chloride exposure. In summary, these *in vitro* studies provide clear evidence that cadmium chloride induces oxidative stress, DNA damage, and programmed cell death in human liver carcinoma (HepG_2_) cells.

## 1. Introduction

Cadmium is one of the naturally occurring heavy metals. However, it is often used in industry, and exerts toxic human health effects. It is classified as a human carcinogen by the International Agency for Research on Cancer and belongs to the group I carcinogens [1]. Cadmium intoxication in humans usually occurs through inhalation (cigarette smoke) and ingestion (consumption of contaminated water and food). Acute intoxication of cadmium may lead to liver, lung, and testis damages [2] while chronic intoxication may result in obstruction of pulmonary disease, disturbance of metabolism, disregulation of blood pressure, obstruction of kidney function, structure of bones and immune system [1,3,4]. Although the mechanism of cadmium induced toxicity is poorly understood, it has been reported that cadmium causes damage to cells through the generation of reactive oxygen species [5]. Studies using two-dimensional gel electrophoresis have shown that several stress response systems are expressed in response to cadmium exposure, including those for heat shock, oxidative stress, stringent response, cold shock, and Son of Sevenless (SOS) [6,7,8]. *In vivo* studies have shown that cadmium modulates male reproduction in a mice model at a concentration of 1 mg/kg body weight [9]. However, cadmium is a weak mutagen when compared with other carcinogenic metals [10]. Previous reports revealed that cadmium affects signal transduction pathways; inducing inositol polyphosphate formation, increasing cytosolic free calcium levels in various cell types [11], and blocking calcium channels [12,13]. A line of evidence shows that cadmium alters antioxidant defense mechanisms and increases generation of reactive oxygen species (ROS) including superoxide anion and hydrogen peroxide [14,15,16]. Hence, the present investigation was designed to prove that oxidative stress plays a key role in cadmium chloride-induced DNA damage and apoptosis of human liver carcinoma (HepG_2_) cells.

## 2. Materials and Methods

### 2.1. Chemicals and Test Media 

DMEM-F12 containing 2.5 mM L-glutamine, 15 mM HEPES, 0.5 mM sodium pyruvate, and 1200 mg/L sodium bicarbonate, was supplied by American Type Culture Collection-ATCC (Manassas, VA, USA), and was used as the growth medium. Costar Company (Cambridge, MA, USA) was the source for obtaining the ninety six-well plates, while Sigma Chemical Company (St. Louis, MO, USA) provided reagents such as fetal bovine serum (FBS), penicillin G and streptomycin, phosphate buffered saline (PBS), G418 and MTT assay kit. 

### 2.2. Cell/Tissue Culture

Human liver carcinoma (HepG2) cells obtained from ATCC were conserved in liquid nitrogen. During experimentation their containers/vials were gently shaken for 2 min in a water bath at 37 °C, and the content of each vial was transferred to a 25 cm^2^ tissue culture flask in which DMEM-F12 medium containing 10% (v/v) fetal bovine serum (FBS), 0.4 mg/mL G418, and 1% (w/v) penicillin/streptomycin, was added up to a total volume of 10 mL. The cells were examined using an inverted tissue culture microscope, and incubated for 24 h in a humidified 5% CO_2_ incubator at 37 °C. The Trypan blue exclusion test (Life Technologies, Carlsbad, CA, USA) was performed to determine the cell viability based on the number of live cells counted, using a hemocytometer.

### 2.3. Assessment of Cell Viability by MTT Assay

HepG_2_ cells were cultured in enriched DMEM-F12 medium as described above, and 180 µL aliquots cell suspension (5 × 10^5^/mL) were pipetted and placed 96-well polystyrene tissue culture plates, followed by the addition of 20 µL aliquots of stock solutions to make-up six replicates of final cadmium chloride concentrations of 1, 2, 3, 4, and 5 µg/mL. Control cells received 20 µL of distilled water. After chemical treatment, HepG2 cells were incubated for 48 h in a humidified 5% CO_2_ incubator at 37 °C. After incubation, the MTT assay for cell viability was performed as previously described [17,18]. 

### 2.4. Assessment of Oxidative Stress by Lipid Hydroperoxide Assay

To test the hypothesis that oxidative stress plays a key role in cadmium chloride-induced toxicity to HepG_2_ cells, lipid hydroperoxide assay (Calbiochem-Novabiochem, San Diego, CA, USA) was performed and the production level of hydroperoxide content was estimated in untreated and treated cells. This experiment was conducted according to the manufacturer’s instructions (Calbiochem-Novabiochem) [19,20], with few modifications as previously described in our laboratory [21,22,23]. 

### 2.5. Assessment of DNA Damage by Comet Assay

The Comet assay was carried out by the method previously described by Collins and his collaborators [24,25] with some modifications [26]. Briefly, 1 × 10^6^ cells/mL were treated with either media or cadmium chloride (0, 1, 2, 3, 4, and 5 µg/mL) respectively and incubated in a 5% CO_2_ at 37 °C for 48 h. After incubation, the cells were centrifuged, washed with cold PBS, and 1 × 10^5^ cells/mL counted from the pool of untreated and treated cells were used for performing the comet assay as previously described [26,27]. 

### 2.6. Assessment of Apoptosis by Annexin V/PI Assay

Annexin V FITC/PI assay was performed as described previously [28] to evaluate the apoptotic effect of cadmium chloride to human liver carcinoma (HepG_2_) cells. Briefly, 2 mL of cells (1 × 10^6^ cells/mL) were added to each well of 6 plates and treated with 1, 2, 3, 4, and 5 µg/mL of cadmium chloride for 48 h. Control well plates were also made without cadmium chloride. After 48 h of incubation, 1 × 10^6^ cells/mL were counted and washed in PBS, re-suspended in binding buffer (10 mm Hepes/NaOH pH 7.4, 140 mm NaCl, 25mm CaCl_2_), and stained with FITC-conjugated annexin V (Pharmingen, Becton Dickinson Co., San Diego, CA, USA). After staining, the cells were incubated for 15 min in the dark at room temperature. Cells were re-washed with binding buffer and analyzed by flow cytometry (FACS Calibur; Becton-Dickinson) using CellQuest software. 

## 3. Results and Discussion

### 3.1. Inhibition of Cell Viability by Cadmium Chloride

**Figure 1:**
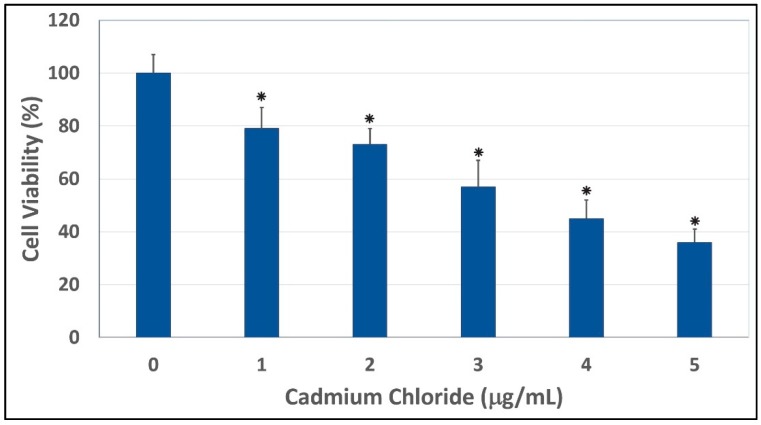
Cytotoxic effect of cadmium chloride on human liver carcinoma (HepG_2_) cells. Cells were cultured with increasing concentrations (1, 2, 3, 4, and 5 μg/mL) of cadmium chloride for 48 h as indicated in the Materials and Methods. Cell viability was determined based on the MTT assay. Each point represents a mean ± SD of three experiments with six replicates per concentration. * Significantly different (*p <* 0.05) from the control, according to the Dunnett’s test.

The results of the cytotoxicity of cadmium chloride to human liver carcinoma (HepG_2_) cells are presented in Figure 1. Data obtained from this assay demonstrated a strong concentration-response relationship with regard to the cytotoxic of cadmium chloride in HepG_2_ cells. As indicated in this Figure, there was a gradual decrease in the viability of HepG_2_ cells, with increasing concentrations of cadmium chloride that resulted in a 48 hr-LD_50_ of 3.6 µg/mL. HepG_2_ cells exposed to cadmium chloride concentrations of 1, 2, 3, 4, and 5 µg/mL showed significant mortalities (*p <* 0.05) compared to control cells, according to ANOVA Dunnett’s test (Figure 1). Cadmium, a potent toxic metal, has a high potential to accumulate in the environment. It is very harmful to the environment and to human beings. The toxicity of cadmium as an industrial pollutant and a food contaminant, and as one of the major components in cigarette smoke is well established [29]. Cadmium can cause a number of lesions in many organs, such as the kidney, the testis, the lung, the liver, the brain, the bone, the blood system [30]. However, the mechanism of toxicity of cadmium is not yet clear. In the present study, we showed that cadmium chloride at the concentrations of 1, 2, 3, 4, and 5 µg/mL significant caused cell mortalities (*p <* 0.05) compared to the control. Consistent with our report, recent study indicated that cadmium chloride is highly cytotoxic to human lens epithelial cells [31]. *In vitro* studies also indicated that cadmium induces cytotoxic effects and free radical-dependent DNA damage in bacteria [32,33], which causes single-strand DNA damage and disrupts the synthesis of nucleic acids and proteins [34]. Published studies reported that acute ingestion of cadmium causes health adverse effects such as abdominal pain, burning sensation, nausea, vomiting, salivation, muscle cramps, vertigo, shock, loss of consciousness and convulsions usually appear within 15 to 30 min [35]. Acute cadmium ingestion can also cause gastrointestinal tract erosion, pulmonary, hepatic or renal injury and coma, depending on the route of poisoning [35,36]. Cadmium can cause a number of lesions in many organs, such as the kidney, the testis, the lung, the liver, the brain, the bone, the blood system [30]. Human exposure to cadmium is possible through a number of several sources including employment in primary metal industries, eating contaminated food, smoking cigarettes, and working in cadmium-contaminated work places, with smoking being a major contributor [1,37]. Other sources of cadmium include emissions from industrial activities, including mining, smelting, and manufacturing of batteries, pigments, stabilizers, and alloys [38]. Cadmium is also present in trace amounts in certain foods such as leafy vegetables, potatoes, grains and seeds, liver and kidney, and crustaceans and mollusks [39].

### 3.2. Induction of Lipid Hydroperoxide by Cadmium Chloride

**Figure 2:**
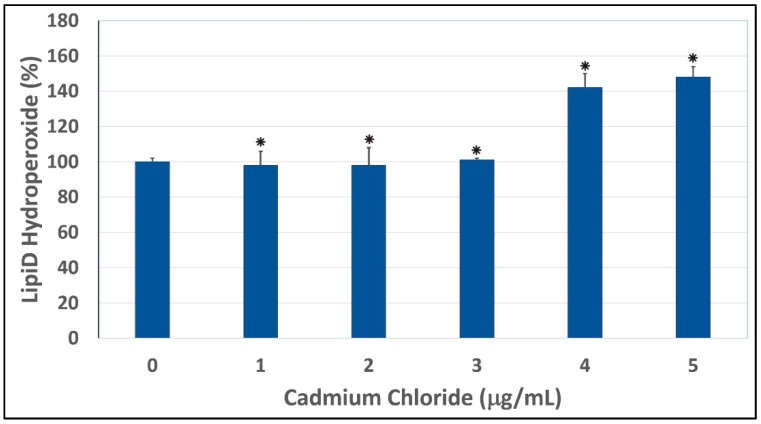
Cadmium chloride-induced lipid peroxidation in human liver carcinoma (HepG_2_) cells. Cells were incubated for 48 h with increasing concentrations of cadmium chloride (1, 2, 3, 4, and 5 μg/mL). Lipid hydroperoxide levels were determined as described in Materials and Methods. * Significantly different (*p <* 0.05) from the control, according to the Dunnett’s test. Data are representative of three independent experiments.

To test whether oxidative stress plays a key role in cadmium chloride-induced toxicity to HepG_2_ cells, we performed a lipid hydroperoxide assay. As shown in Figure 2, our results indicated that the treatment of HepG_2_ cells with cadmium chloride resulted in a significant increase of lipid hydroperoxide levels, a major degradation product of unsaturated phospholipids and glycolipids. Similar results have been recorded by Bashandy and collaborators [40]. Our result is also in agreement with a previous report indicating that cadmium induces formation of superoxide ion and hydrogen peroxide in HeLa human tumor cells and bovine aorta endothelial cells [41]. Consistent with our result, previous reports also indicated that lead and cadmium-induced tissue damages through oxidative stress [42]. Cadmium stimulates the formation of metallothioneins and reactive oxygen species, thus causing oxidative damage to erythrocytes and various tissues resulting in loss of the membrane functions [43]. Another study indicated that chronic exposure to cadmium increased lipid peroxidation and caused inhibition of superoxide dismutase (SOD) activity showing oxidative damage in liver, kidney, and testes [44]. A previous scientific report showed that treatment with cadmium causes a more pronounced reduction in intracellular glutathione levels and a significantly higher free radical accumulation in progenitors [45]. Other reports indicated that cadmium decreases intracellular glutathione content and activities of cellular antioxidant enzymes, superoxide dismutase, peroxidase and catalase, leading to the accumulation of ROS and an increase in intracellular oxidative stress in cadmium exposed CRL-1439 normal rat liver kidney cells [46,47]. 

### 3.3. Induction of DNA Damage by Cadmium Chloride

**Figure 3:**
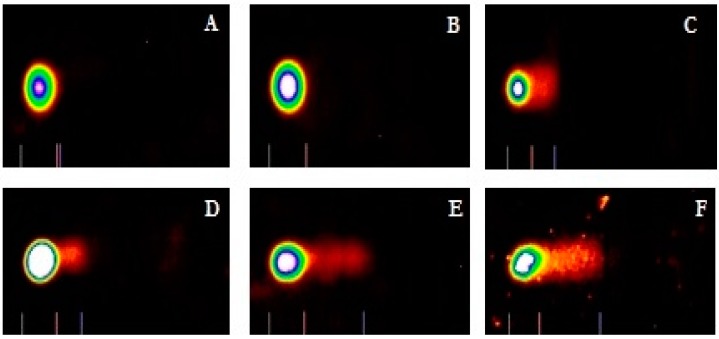
Cadmium chloride induced DNA damage in human liver carcinoma (HepG_2_) cells. Cells were treated for 48 hours with medium (**A**) supplemented with solvent or 1 (**B**); 2 (**C**); 3 (**D**); 4 (**E**); and 5 (**F**) μg/mL cadmium chloride. Representative comet images were analyzed using LAI’s Comet Assay Analysis System software (Loates Associates, Inc. Westminster, MD, USA).

**Figure 4:**
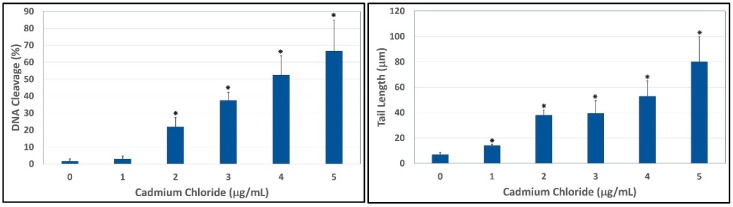
Comet assay of HepG_2_ cells showing the percentages of DNA cleavage (**Left**) and comet tail lengths (**Right**) as a function of cadmium chloride concentrations. Each point represents mean ± SD of three independent experiments. * Significantly different (*p <* 0.05) from the control, according to the Dunnett’s test.

To evaluate the ability of cadmium to trigger genotoxic damage in hepatocytes, HepG_2_ cells were treated with different concentrations of cadmium chloride, in the range 0–5 μg/mL for 48 h, and the degree of DNA damage was quantified by the means of LAI’s Comet Assay Analysis System software (Loates Associates, Inc. Westminster, MD, USA) after staining with SYBR Green. Our results showed that cadmium chloride at the concentrations of 1, 2, 3, 4 and 5 μg/mL causes DNA single strand breaks in HepG_2_ cells and there is a gradual concentration-response relationship. The representative comet assay images of control and cadmium chloride-treated HepG_2_ cells are presented in Figure 3. This Figure showed a significant increase in the percentage of DNA damage and length of comet tail in a concentration-dependent manner in human liver carcinoma cells exposed to cadmium chloride. The percentages of DNA cleavage were as follows: (1.7 ± 1.2)%, (2.9 ± 1.6)%, (21.9 ± 5.5)%, (37.5 ± 4.7)%, (52.4 ± 11.4)%, and (66.5 ± 18.3)% for 0, 1, 2, 3, 4 and 5 µg/mL of cadmium chloride, respectively (Figure 4). Similar trend was observed in the mean length of comet tail. The tail lengths of DNA comets were all longer in cadmium chloride-treated cells compared to the control (*p* < 0.05) (Figure 4). 

Several possible mechanisms may be involved in the induction of DNA damages. With the currently available data, cadmium chloride seems to have direct genotoxic activity to HepG_2_ cells at concentrations relevant tested. Studies have provided evidence that reactive oxygen species (ROS) are involved in DNA damage induced by carcinogenic metal ion [48]. It has been shown that cadmium enhances lipid peroxidation in cultured cells and animals [49,50]. Low concentrations of cadmium binds to proteins, decreases DNA repair [51], activates protein degradation, up-regulates cytokines and proto-oncogenes such as c-*fos*, c-*jun*, and c-*myc* [52], and induces expression of several genes including metallothioneins [53], heme oxygenases, glutathione transferases, heat-shock proteins, acute-phase reactants, and DNA polymerase β [54]. 

### 3.4. Induction of Apoptosis by Cadmium Chloride

**Figure 5:**
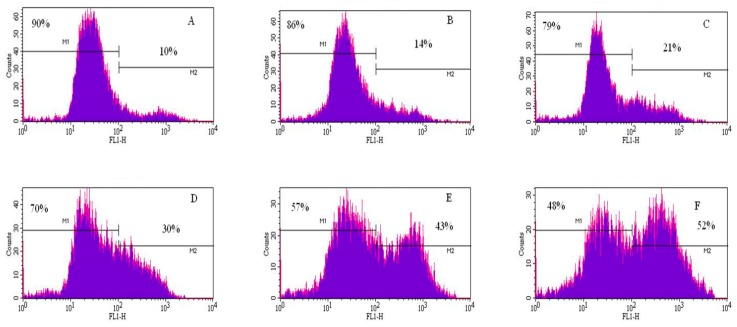
Representative flow cytometry analysis data from annexin V/PI assay. The histograms show a comparison of the distribution of annexin V/PI negative cells (M1) and annexin V/PI positive cells (M2) after 48 h exposure to cadmium chloride. **A** = control; **B** = 1 μg/mL; **C** = 2 μg/mL; **D** = 3 μg/mL; **E** = 4 μg/mL; and **F** = 5 μg/mL.

**Figure 6:**
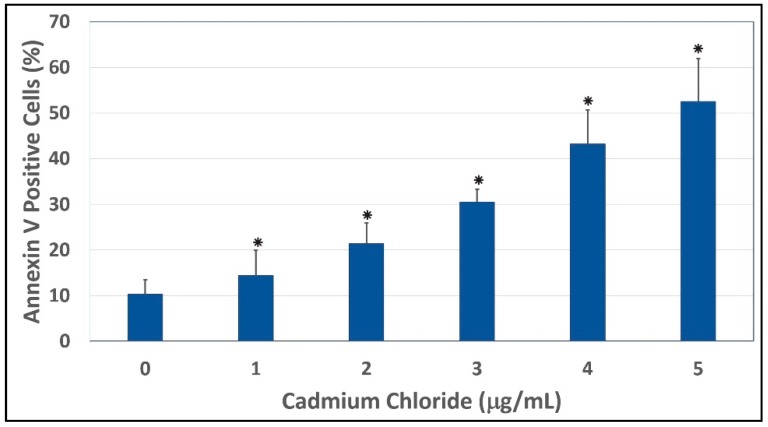
Annexin V and PI positive cells. Cells were exposed to different concentrations of cadmium chloride as described in the Materials and Methods. * Significantly different (*p <* 0.05) from the control, according to the Dunnett’s test.

To gain insight into the mechanism of cadmium chloride-induced apoptosis, we examined the modulation of phosphatidylserine externalization in HepG_2_ cells. We observed that cadmium chloride induces cellular apoptosis of HepG_2_ cells in a concentration-dependent manner, showing a gradual increase of annexin positive cells in cadmium chloride-treated cells compared to the control (Figure 5). Figure 6 shows the percentages of both annexin V and PI positive cells were (10.3 ± 3.2)%, (14.4 ± 5.6)%, (21.4 ± 4.6)%, (30.5 ± 2.8)%, (43.2 ± 7.5)%, and (52.5 ± 9.4)% in 0, 1, 2, 3, 4, and 5 µg/mL cadmium chloride, respectively. 

The effect of cadmium chloride was more pronounced at 5 μg/mL (*p* *<* 0.05) compared to the control cells. We observed that the percentage of annexin positive cells increased gradually (*p* *<* 0.05) with increasing cadmium chloride concentrations and reached a maximum of (52.5 ± 9.4)% cell death upon 48 h of exposure. Cadmium can affect cell proliferation and differentiation, cell cycle progression, DNA synthesis and repair, apoptosis and other cellular activities [55,56]. Recently, several reports have shown that cadmium can induce apoptosis of many tissues and cells both *in vivo* and *in vitro* including cells of the respiratory system, the testis, the kidney, the liver, and the immune system [57,58,59,60,61]. 

In addition to the apoptotic effects of cadmium, other reports have indicated that cadmium chloride decreases the viability of HepG2 cells and increases lactate dehydrogenase leakage, DNA damage, malondialdehyde, and antioxidant enzymes activities [62,63]. Additionally, a significant decrease in ATP production and increase in ROS levels in cadmium chloride-treated HepG2 cells was observed at all concentrations tested. A significant decrease in GSH/GSSG ratio was also found. These effects were reported to be attenuated when cells were co-exposed to *N*-acetylcysteine (NAC) and cadmium chloride [62,63].

## 4. Conclusions

The present *in vitro* study demonstrated that cadmium chloride exposure gradually decreases the viability of HepG_2_ cells; increases lipid hydroperoxide levels resulting from reactive oxygen species formation; induces DNA damage and triggers apoptosis of HepG_2_ cells through phosphatidylserine externalization. In summary, these findings suggest that oxidative stress plays a role in cadmium chloride-induced cyto/genotoxicity and apoptosis of HepG_2_ cells, especially at higher level of exposure (4 and 5 μg/mL) where CdCl_2_-treated cells show a statistically significant difference in lipid hydroperoxide concentrations compared to control cells. This study therefore provides insight into the mechanism underlying cadmium chloride-induced toxicity and apoptosis of HepG_2_ cells. 
